# Massive Subchorionic Thrombohematoma (Breus' Mole) Associated with Fetal Growth Restriction, Oligohydramnios, and Intrauterine Fetal Death

**DOI:** 10.1155/2019/9510936

**Published:** 2019-04-30

**Authors:** Miyuki Miyagi, Tadatsugu Kinjo, Keiko Mekaru, Hayase Nitta, Hitoshi Masamoto, Yoichi Aoki

**Affiliations:** Department of Obstetrics and Gynecology, Graduate School of Medicine, University of the Ryukyus, 207 Uehara Nishihara, Okinawa 903-0215, Japan

## Abstract

Massive subchorionic thrombohematoma (MST), termed Breus' mole, is a rare condition in which a large maternal blood clot separates the chorionic plate from the villous chorion. Common complications of MST include fetal growth restriction, preeclampsia, and intrauterine fetal death. Here, we present a case of a 17-year-old Japanese woman referred to our institution at 21 weeks of gestation. Ultrasound examination revealed a large placental mass with mixed high and low echogenicity measuring approximately 7.6 cm in thickness. Doppler examination showed absence of end-diastolic velocity of the umbilical artery. At 22 weeks of gestation, the patient had a stillbirth weighing 138g. The placenta weighed 502 g and was 8 cm thick, and the total blood loss was 270 g. Macroscopic examination revealed that a subchorionic blood clot measuring 12 cm × 5 cm covered a large portion of the placenta with well-defined margins on the fetal surface. Microscopic examination revealed an intervillous hematoma and fibrinous deposits directly beneath the chorionic plate with adjacent compressive effects. Based on these findings, MST was diagnosed. Because MST is rare, it must be considered in the differential diagnosis of parental conditions. Magnetic resonance imaging can be optimal for diagnosing MST when ultrasound diagnosis is difficult.

## 1. Introduction

Massive subchorionic thrombohematoma (MST) is a rare condition in which a large maternal blood clot separates the chorionic plate from the villous chorion. It was first described and termed Breus' mole in 1892 [1]. MST is usually complicated by other serious conditions, including fetal growth restriction (FGR), preeclampsia, and intrauterine fetal death (IUFD). The incidence of MST is only 0.03%–0.08% [2, 3], and its etiology and pathogenesis are yet unknown. The outcomes of MST depend on its size and site. If MST occurs near the cord insertion, it can cause cord compression, umbilical venous obstruction, and decreased fetal perfusion. Therefore, MST is associated with complications such as FGR or IUFD caused by placental insufficiency [3, 4].

Here, we report a rare case of MST that presented with placentomegaly and complicated by FGR and oligohydramnios at 21 weeks of gestation.

## 2. Case Presentation

**Figure 1:**
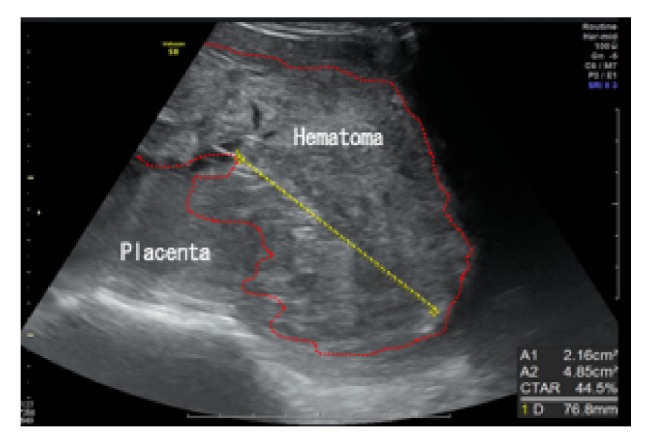
Ultrasound examination revealed a large placental mass with mixed high and low echogenicity measuring approximately 7.6 cm in thickness. The range of the presumed hematoma is indicated by the dotted lines.

**Figure 2:**
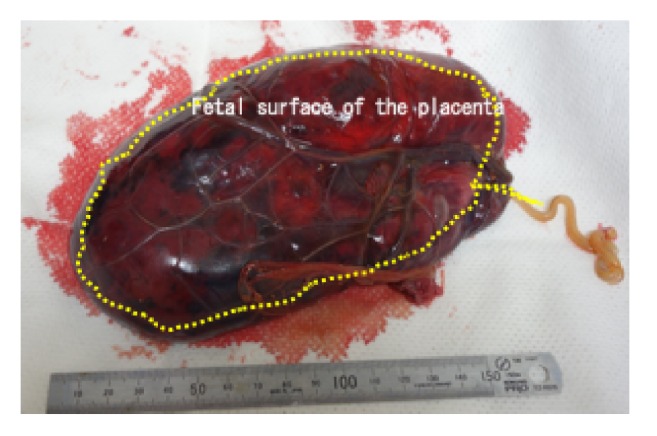
A subchorionic blood clot measuring 12 cm × 5 cm covered a large portion of the placenta and had well-defined margins on the fetal surface (shown by dotted lines).

**Figure 3:**
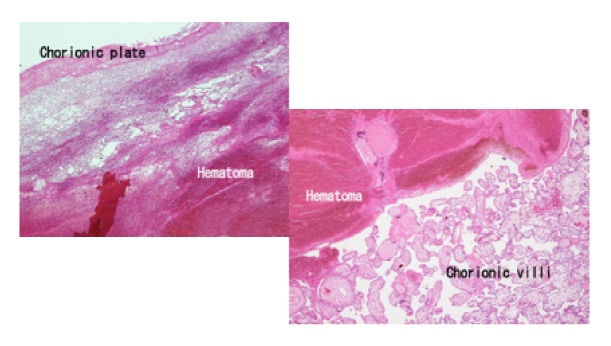
Microscopic examination revealed an intervillous hematoma and fibrinous deposits directly beneath the chorionic plate with adjacent compressive effects (hematoxylin-eosin staining, 20× magnification).

A 17-year-old Japanese woman, para 0, was referred to our institution at 21 weeks and 6 days of gestation following the observation of FGR, oligohydramnios, and placental enlargement which were observed at a private clinic. Ultrasound (Figure 1) revealed a large placental mass with mixed high and low echogenicity. The reason underlying the enlargement of the placenta was unclear; therefore, we only measured its thickness, which was approximately 7.6 cm. We observed FGR with the following fetal biometry measures: biparietal diameter (BPD) of 31.9 mm (−6.9, standard deviation [SD]), abdominal circumference of 102.3 mm (−5.1, SD), femur length of 20.7 mm (−5.0, SD), and estimated fetal body weight of 100 g (−4.9, SD). Furthermore, severe oligohydramnios was observed, and Doppler examination revealed the absence of end-diastolic velocity (AEDV) of the umbilical artery. Dextroposition of the fetal heart was observed. Maternal serum analyses showed no TORCH syndrome. The cervical length was 8 mm, and the patient was admitted for tocolysis. However, she experienced labor pains on the following day and had a stillbirth weighing 138 g with low set ears (gender unknown) at 22 weeks of gestation, with a total blood loss of 270 g. The placenta weighed 502 g and was 8 cm in thickness. Macroscopic examination revealed that a subchorionic blood clot measuring 12 × 5 cm covered a large percentage of the placenta with well-defined margins on the fetal surface (Figure 2). A large amount of blood was stored in the subchorionic space. No hematoma or infarction was observed on the maternal surface of the placenta. Microscopic examination revealed an intervillous hematoma and fibrinous deposits directly beneath the chorionic plate with adjacent compressive effects (Figure 3). Based on these findings, MST was diagnosed. FGR and dextroposition of the fetal heart were observed by ultrasound, and low set ears were observed after birth. We recommended chromosomal analysis and autopsy of the fetus; however, the patient and her husband denied these. The patient had an uneventful postoperative course.

## 3. Discussion

In the present case, ultrasound revealed placentomegaly with mixed high and low echogenicity, FGR, and severe oligohydramnios. Doppler examination revealed AEDV of the umbilical artery. However, prenatal diagnosis of MST could not be performed because of severe oligohydramnios.

Ultrasound findings of MST have revealed extensive echogenic areas near the chorionic plate, indicating hemorrhage, which differs from normal placental tissue. Fung et al. [3] reported that MST may appear to be a heterogeneous, homogenous, or hypoechogenic mass in the chorion, which is distinct from the normal ultrasonic texture of placental tissue, and can present as placentomegaly. With regard to a case of suspected placenta previa, elastography was reported to clearly differentiate a hematoma from the placenta [5]. However, prenatal diagnosis using ultrasound is often challenging. In our case, diagnosis of MST by ultrasound was not possible because of severe oligohydramnios.

Although magnetic resonance imaging (MRI) findings of MST are variable, MRI is reportedly useful for differentiating MST from other placental diseases, such as placental abruption, chronic abruption oligohydramnios sequence (CAOS), and placental mesenchymal dysplasia (PMD) [6, 7]. In a previous case, MRI revealed a large mass between the placental parenchyma and amniotic cavity and a slightly and partially high signal on T2- and T1-weighted images inside the mass, respectively. Thrombohematoma was represented by a low signal at the marginal zone on both T1- and T2-weighted images and a high signal at the rim on T1-weighted images. Moreover, steady-state free precession (SSFP) MRI has been used to clearly show the arrangement of the hematoma, placenta, umbilical cord, and fetus [6]. In a previous report, 9 of 14 subchorionic hematomas were confirmed using MRI [7]. Overall, these findings suggest that MRI is optimal for diagnosing MST when ultrasound diagnosis is difficult. Accordingly, MRI was planned for our patient; however, she had a stillbirth at 22 weeks of gestation before MRI was performed.

The etiology of MST remains obscure. In MST, a large amount of blood, particularly of maternal origin, collects and separates the chorionic plate from the villous chorion. DNA analysis has revealed that 85% of the blood in the thrombus is maternal in origin [8]. Fetal villous hemorrhage or obstruction with subsequent accumulation of blood and massive separation of the chorionic plate has been proposed to cause MST [9]. Furthermore, stasis of maternal blood in the subchorionic space results in thrombosis [3, 4]. Recently, MST has been reported in patients with thrombophilic conditions [10] and following thrombolytic therapy [11].

Uteroplacental insufficiency is the underlying mechanism of FGR. In cases of severe FGR, at least half of placental surface might be covered with hemorrhage under the chorionic plate [12]. In such cases, the finding of normal UA Doppler waveforms at presentation is a favorable prognostic sign of perinatal survival [4]. Furthermore, in MST, the site of major hemorrhage is important whether it resides entirely within the membranes and distant from the placental disc or detach placental implantation [12].

MST is associated with poor pregnancy outcomes. Fung et al. [3] reported that only 6 of 10 pregnancies with MST resulted in a live birth, only 2 of which reached full term. Alanjari et al. [4] compared seven survivors and seven nonsurvivors with MST and reported that MST can be diagnosed in the second trimester by ultrasound examination of the placenta. In addition, they reported that normal fetal growth and normal umbilical artery Doppler waveforms were significantly associated with perinatal survival [4].

Here, we reported a case of MST that presented with placentomegaly complicated by FGR and oligohydramnios. Because of severe oligohydramnios, prenatal diagnosis of MST in the patient was not possible. Nevertheless, MST must be considered in the differential diagnosis of parental conditions because it is a rare condition. Furthermore, this case highlights the potential use of MRI for diagnosing MST when ultrasound diagnosis is difficult.
